# Fine-Scale Landscape Epidemiology: Sarcoptic Mange in Bare-Nosed Wombats (*Vombatus ursinus*)

**DOI:** 10.1155/2023/2955321

**Published:** 2023-03-04

**Authors:** Leah G. Burgess, Shane A. Richards, Michael M. Driessen, Vicky Wilkinson, Rahil J. Amin, Scott Carver

**Affiliations:** ^1^School of Natural Sciences, University of Tasmania, Hobart, Tas 7001, Australia; ^2^Department of Natural Resources and Environment Tasmania, Hobart, Tas 7001, Australia

## Abstract

Landscape epidemiology provides a valuable framework to interpret, predict, and manage spatiotemporal patterns of disease. Yet, owing to the difficulty of detecting pathogen occurrence in free-ranging wildlife, disentangling the factors driving disease dynamics remains a considerable challenge, particularly at fine spatial scales. Here, we investigated the fine-scale landscape epidemiology of sarcoptic mange—a visually apparent disease caused by the mite *Sarcoptes scabiei*—in bare-nosed wombats (*Vombatus ursinus*), by: (1) characterizing the distribution and density of wombats within the landscape and (2) examining the effect of environmental variation on the occurrence and apparent prevalence of mange. Wombats were heterogeneously distributed over 19.4 km of transect space (0.096–1.39 wombats ha^−1^) and seven months of time (increasing by a factor of 1.76). Wombat density was negatively associated with distance to vegetation cover, supporting a general propensity for wombats to occur and burrow near vegetation (native and exotic, excluding pasture). The apparent prevalence of mange varied spatially (3.1–37.5%), with the probability of disease greater in wombats with minimal vegetation and low-lying pans in their estimated home range. We observed trends of increased prevalence in areas with more burrows available per wombat and in individuals occurring near vegetation cover (although not within their home range). Wombat density and active burrow density did not influence the prevalence of mange. This research emphasizes the fine scale at which spatiotemporal patterns of disease can manifest and is the first to investigate the influence of host density for any species with indirect transmission of *S. scabiei*. Collectively, our results suggest that individuals inhabiting less optimal habitat (pasture) may be at greater risk of disease, or that diseased wombats may be competitively excluded from more optimal habitat (vegetated areas). We discuss implications for understanding and managing mange in wombats and cross-applicability to other mange-affected species with environmental transmission.

## 1. Introduction

Landscape epidemiology (a.k.a., spatial epidemiology) seeks to describe and understand spatiotemporal variation in infectious disease risk or incidence, with respect to the underlying environment [1, 2]. The field stems from recognition that many epidemiological processes are driven by environmental conditions (both biotic and abiotic) that influence the distribution and dynamics of host, vector, and pathogen populations [3, 4]. By this nature, landscape epidemiology provides a particularly valuable framework to better understand and potentially manage pathogens affecting wildlife, as wildlife are typically distributed across gradients of environmental conditions. This understanding is commonly achieved by using Geographic Information System (GIS) tools to construct disease distribution maps overlain with georeferenced environmental data and assuming those conditions with the greatest degree of spatial concordance to be causative [1]. In addition, GIS and innovative analytical methods can be used to project the distribution of identified conditions to other geographic regions or times and provide an *a priori* understanding of disease risk or spread [3]. However, owing to the difficulty of detecting and documenting pathogen occurrence in free-ranging wildlife, disentangling the factors driving spatiotemporal patterns of disease remains a considerable challenge, particularly at fine scales that require continuous sampling in space [5, 6].

Sarcoptic mange (hereafter “mange”) is a contagious skin disease caused by infection with the parasitic mite, *Sarcoptes scabiei*. The pathogen is of global significance, present on all continents (except Antarctica) and documented in approximately 150 species across 12 mammalian orders, including humans, domestic animals, and wildlife [7]. Infection typically results in type I (immediate) or type IV (delayed) hypersensitivity reactions, characterized by a range of highly visible signs, such as alopecia, hyperkeratosis, emaciation, and pruritus [8, 9]. Therefore, unlike many wildlife diseases that require laboratory diagnosis, mange can be diagnosed non-invasively via visual assessment and is thus more readily documented in free-ranging wildlife [10, 11]. Among its broad and expanding host range, *S. scabiei* maintains transmission via three general mechanisms: direct transmission between hosts, indirect (environmental) transmission via pathogen-carrying fomites, and combined direct and indirect transmission [12]. The emergence, spread, and persistence of mange among wildlife are fundamentally driven by biotic (e.g., host density and behavior) and abiotic (e.g., microclimate and landscape structure) factors that facilitate interindividual contact and/or off-host* S. scabiei* survival [7].

Mange is the most significant and deleterious infectious disease of the bare-nosed wombat (*Vombatus ursinus;* a.k.a., common wombat), typically persisting endemically throughout their geographic range with periodic epizootics causing localized population decline and extirpation events [13, 14]. Following infection, wombats typically suffer from crusted mange—characterized by a type IV hypersensitivity reaction—and experience severe morbidity and mortality [11, 15]. Intraspecies transmission occurs predominately through environmental exposure, as wombats are largely non-territorial and rarely come into direct contact outside of mating [16]. Wombat burrows are believed to act as environmental reservoirs and the sites responsible for most transmission events, as wombats switch burrows every 4–10 days and engage in asynchronous burrow sharing [17, 18]. This assumption is further supported by evidence that within-burrow microclimatic conditions are relatively uniform and suitable to sustain off-host* S. scabiei* survival for 6–16 days, depending on season [19].

Disease models of the wombat-mite system suggest that a wombat population can exhibit one of four epidemiological outcomes [20, 21]. These are as follows: (1) total extinction of the wombat and mite populations; (2) isolated extinction of the mites; (3) an unstable coexistence with repeated epidemics, with the wombat population reduced well below environmental carrying capacity; or (4) an enzootic equilibrium, with the wombats held below carrying capacity. Empirical evidence suggests that endemic disease (outcome 4) is the most common scenario in which mange occurs in wombat populations [22, 23]. However, the causal mechanisms and landscape factors shaping disease risk in this scenario remain poorly understood, particularly at fine spatial scales [20]. Furthermore, the influence of host density has not been empirically investigated for any species with indirect transmission of this parasite [12], despite the seemingly obvious potential for density to alter environmental exposure rates.

Here, we examine the fine-scale landscape epidemiology and risk factors associated with a visually apparent, endemic disease. We have two objectives: (1) to characterize the geographic distribution and abundance of wombats within the landscape and (2) to investigate the effect of environmental variation on the occurrence and apparent prevalence (i.e., proportion of observed wombats exhibiting visible signs of disease) of sarcoptic mange. Given that intraspecies transmission is predominately indirect and occurs via contaminated burrows [17], we hypothesize that the occurrence of mange will largely be driven by the availability of burrows in the landscape and fine-scale abiotic factors that influence off-host *S. scabiei* survival. In addition, as wombats switch burrows periodically with varying delays between occupancy [24], we hypothesize greater transmission potential via increased burrow sharing in more densely populated areas. Collectively, this research will contribute to understanding fine-scale spatiotemporal variation in *S. scabiei* exposure and has important implications for predicting future disease spread and implementing effective control interventions.

## 2. Materials and Methods

### 2.1. Study Site

**Figure 1:**
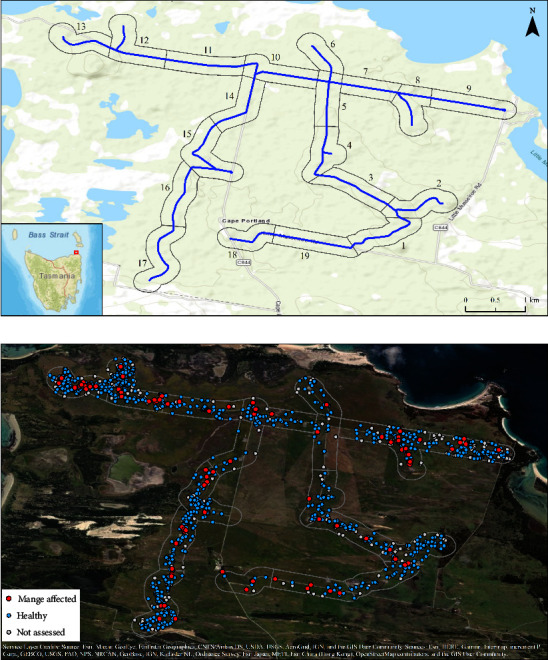
Map showing (a) location of the study area at Musselroe Wind Farm, Cape Portland, with the 19.4 km observational survey route (blue) and 19 ∼1 km road sections (grey) and (b) geographic location of all affected (red; *n* = 95), healthy (blue; *n* = 914), and not assessed (white; *n* = 337) wombats observed during the study period.

We conducted this research at Musselroe Wind Farm, Cape Portland, Tasmania (Figure 1(a)), owing to the presence of a high-density wombat population and endemic mange [22]. The site is characterized by extensive areas of introduced pasture which are grazed for beef production. Remnant vegetation communities generally occur close to the coast and comprise: *Acacia longifolia* coastal scrub, *Allocasuarina verticillata* forest, coastal grass and heathland, and saline aquatic herbfield [25]. The site is mostly low-lying (<20 m asl), poorly drained and formed on Quaternary siliceous marine sands and clays [25, 26]. Mean annual rainfall at Cape Portland is 733 mm, with 50–77 mm falling each month, excluding February which receives an average of 35 mm [27]. Average temperatures range between 14.6°C and 20.6°C in summer and 8.7°C and 13.6°C in winter [27].

### 2.2. Host and Disease Surveys

To estimate wombat density and the apparent prevalence of mange, we undertook eight day (between 11 : 00 and 17 : 00) and night (commencing shortly after civil dusk) vehicle-based observational surveys along a 19.4 km transect (Figure 1(a)), between the months of March and September in 2021. When a wombat was sighted, the vehicle was stopped and experienced observers visually examined the wombat for evidence of mange—primarily through signs of alopecia and hyperkeratosis (Table S1)—using 10 by 42 binoculars and a hand-held 1,200 lumen spotlight (Powa Beam Meteor MS1) at night. Wombats that disappeared out of view too quickly or were obscured by vegetation were not assessed for mange (25% of observations, see results). For each observation, we recorded the GPS location of the vehicle on the transect, as well as a compass bearing and estimate of distance from the vehicle to the wombat (0–10, 10–25, 25–50, 50–75, 75–100, or >100 m). Care was taken to ensure that each wombat was not counted more than once within a survey, although a subset of individuals was likely observed across multiple surveys.

### 2.3. Burrow Density

We estimated burrow density and the ratio of burrows available per wombat, as intraspecies transmission predominately occurs via asynchronous burrow sharing [17]. Burrow density estimates were made for 19 ∼1 km length sections of 150 m in width (estimated observable area; see Figure 1(a)). We systematically surveyed a minimum of three 50 by 50 m (some initially larger) quadrats per section for burrows (Figure S1A) and assigned each an activity status: active (i.e., fresh scats, diggings, and lack of vegetation overgrowth at the burrow entrance), recently active (i.e., old scats and general lack of overgrowth at the entrance), or inactive (i.e., overgrown, collapsed, and flooded burrows). We calculated a mean active burrow density (burrows ha^−1^) and an active burrow to wombat ratio for each section, presuming active burrows to be facilitative of transmission.

### 2.4. Landscape Covariates

We collected data on environmental features considered potentially important for predicting the local-scale distribution of wombats and off-host *S. scabiei* survival. We generated a feature class layer in ArcMap (ESRI®) at a 1 : 3,000 scale using a satellite image of the study area and drawing polygons around eight identifiable features: roads, wind turbines, buildings, introduced pasture, remnant vegetation (native and exotic, excluding pasture), water bodies, low-lying pans, and areas of sand (Figures S2A and S2B). We then rasterized the feature layer, stratified each road segment into 10 by 10 m grids, and extracted the most frequent feature type overlaying each cell. We also repeated this at the individual wombat scale, using ∼10 ha circular buffers around each observation—representing an approximate midpoint between average wombat home range and core area sizes [24]—to account for wombat movement. We then calculated the proportion of the eight aforementioned features comprising each road segment and potential home range.

In addition, we calculated a minimum straight-line distance to vegetation cover for each road segment using ArcMap (ESRI®) (Figure S1B). Cover was identified from a satellite image as large areas of dense vegetation—generally occurring in the surrounding areas of the site—that were presumed to provide wombats with refuge and contain a high density of wombat burrows [28]. This is distinct from the proportion of remnant vegetation as defined previously, which included all vegetation contained within each road segment or wombat home range, but was generally not dense.

### 2.5. Analyses

#### 2.5.1. Predicting Wombat Density

Wombat densities were estimated for each of the 19 road segments using distance sampling [29] and Bayesian methods [30]. Following preliminary data explorations, we omitted “road,” “wind turbine,” “sand,” “building,” and “waterbody” owing to low site coverage (<5%), and “introduced pasture” and “low-lying pan” owing to high correlation with each other and “remnant vegetation” (*r* > 0.8). “Remnant vegetation” was retained within the model as it displayed high inter-road variation (0.23–32.5%) and was regarded as the landscape feature most likely to influence wombat occurrence and *S. scabiei* persistence [28, 31]. Therefore, we considered four fixed effects: survey date, minimum distance to vegetation cover, proportion of remnant vegetation, and active burrow density. We included “survey trip” and “road section” as random effects within the model to account for unmeasured variation.

The model assumed that wombat density (wombats ha^−1^) in road section *j* and on survey *k*, denoted by ${\bar{\lambda }}_{jk}$, had the form as follows:

(1) $$\begin{array}{c} {\bar{\lambda }}_{jk}=\exp\;\left(\alpha_{0}+\overset{M}{\underset{m=1}{\sum }}\alpha_{m}z_{m}+\delta_{j}+\varepsilon_{k}\right)\text{,} \end{array}$$

where road section *j* is associated with the *M* = 4 landscape covariates, *x* = {*x*_1_, ⋯, *x*_*m*_}, and *z*_*m*_ is the z-transformation of *x*_*m*_ (to assist with model convergence). The parameters *α*_*m*_ describe the effect of covariate *m* on wombat density. *δ*_*j*_ and *ε*_*k*_ denote the section and survey differences in wombat densities and are assumed to come from a normal distribution with mean zero and standard deviation *σ*_*S*_ and *σ*_*T*_, respectively. Thus, exp (*α*_0_) gives the overall mean wombat density.

Setting the probability a wombat observed at distance *w* as *g*(*w*), the probability an animal is observed, if it is in distance band *i*, is given by:

(2) $$\begin{array}{c} p_{i}=\frac{1}{d_{i}-d_{i-1}}\int_{w=d_{i-1}}^{d_{i}}g\left(w\right)dw\text{,} \end{array}$$

where *d*_*i-*1_ and *d*_*i*_ are the lower and upper distances from the road associated with the band. Assuming a half-normal detection function,

(3) $$\begin{array}{c} g\left(w\right)=\exp\left({-\frac{1}{2}\left(\frac{w}{\sigma_{D}}\right)}^{2}\right), \end{array}$$

implies:

(4) $$\begin{array}{c} p_{i}=\sqrt{\frac{\pi }{2}}\frac{\sigma_{D}}{\left(d_{i}-d_{i-1}\right)}\left[\mathrm{erf}\left(\frac{d_{i}}{\sqrt{2}\sigma_{D}}\right)-\mathrm{erf}\left(\frac{d_{i-1}}{\sqrt{2}\sigma_{D}}\right)\right]\text{,} \end{array}$$

where erf(x) is the error function.

Assuming wombats were observed on both sides of the transect, we calculated the expected number of wombats observed in road section *j* on survey trip *k*, within distance category *i*, using the following equation:

(5) $$\begin{array}{c} {\bar{n}}_{ijk}=p_{i}2l_{j}{\bar{\lambda }}_{jk}\left(z_{m}\right)\left({d_{i}-d}_{i-1}\right)\text{,} \end{array}$$

where *l*_*j*_ is the length of road segment *j*. This can be rewritten as follows:

(6) $$\begin{array}{c} {\bar{n}}_{ijk}=\sqrt{2\pi }\sigma_{D}l_{j}\exp\;\left(\alpha_{0}+\overset{M}{\underset{m=1}{\sum }}\alpha_{m}z_{m}+\delta_{j}+\varepsilon_{k}\right)\left[\mathrm{erf}\left(\frac{d_{i}}{\sqrt{2}\sigma_{D}}\right)-\mathrm{erf}\left(\frac{d_{i-1}}{\sqrt{2}\sigma_{D}}\right)\right]\text{.} \end{array}$$

Variation in wombat numbers about this expectation was assumed to be consistent with a negative-binomial distribution, with a variance to mean ratio of 1+*ϕ* [32].

Diffuse prior distributions were assumed for all model parameters, such that parameter uncertainty was dominated by the data and not the prior. The statistical model was coded using the Stan programming language [33] in R v4.1.1 [34]. Markov Chain Monte Carlo (MCMC) methods were used to estimate the posterior distributions of the parameters. We analyzed two chains of length 1,500 iterations for each parameter with a burn-in period of 500 iterations and confirmed convergence following Gelman and Hill [35]. Credible intervals were used to assess the importance of predictors.

#### 2.5.2. Modeling Patterns of Mange Occurrence

To identify environmental factors associated with the occurrence of mange, we fit a model to our wombat observations where we could confidently assess for evidence of disease. For each animal, we modeled the probability of it being “mange affected,” *π*_*jk*_, by incorporating the following fixed effects: survey date, minimum distance to vegetation cover, proportion of remnant vegetation (within a potential home range), active burrow density, wombat density (wombats ha^−1^, estimated from previous analysis 2.5.1), and the ratio of active burrows to wombats. All fixed effects were z-transformed to assist model convergence. We also included “survey trip” and “road section” as random effects to account for unmeasured variation. The logistic-transformed probability of a wombat being “mange affected” was given by:

(7) $$\begin{array}{c} logit\left(\pi_{jk}\right)=\beta_{0}+\overset{M}{\underset{m=1}{\sum }}\beta_{m}z_{m}+\delta_{j}+\varepsilon_{k}\text{,} \end{array}$$

where logit(*π*) = ln   (*π*/(1 − *π*)), *β*_0_ and *β*_*m*_ are the intercept and slope associated with fixed predictor variable *m*, respectively, and *z*_*m*_ is the z-transformed predictor variable *m*. Like before, *δ*_*j*_ and *ε*_*k*_ describe the road section and survey differences and are drawn from a normal distribution with mean zero and standard deviation *σ*_S_ and *σ*_T_, respectively.

Model parameter uncertainty was again estimated using Bayesian methods; however, for this simpler model (i.e., did not require estimation of a detection curve), we used the stan-glmer function provided by the rstanarm package [30]. Again, we used diffuse prior distributions for all parameters included in the model, considered two chains of length 1,500 iterations with a burn-in period of 500 iterations and confirmed convergence following Gelman and Hill [35].

## 3. Results

**Figure 2:**
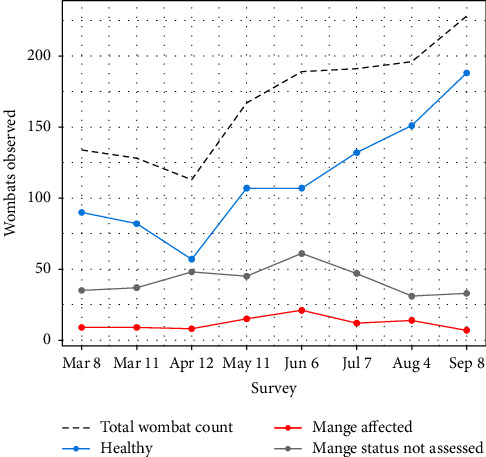
Changes in wombat count and mange status at Musselroe Wind Farm, Cape Portland, across eight observational surveys.

Over seven months, we performed eight observational survey trips (day and night surveys combined) and made 1,346 wombat observations. We assessed 1,009 observed wombats for visible signs of mange and diagnosed disease in 95 (Figure 1(b)). Wombats and mange were found to be widespread across the site, with at least one mange-affected wombat observed in each section throughout the survey period. Wombat counts varied considerably across the surveys, ranging between 113 and 228, displaying a generally-positive temporal trend (Figure 2). The number of mange-affected wombats observed per survey varied between seven and 21, with no clear temporal trend detected (Figure 2).

### 3.1. Wombat Detection and Predictors of Density

**Figure 3:**
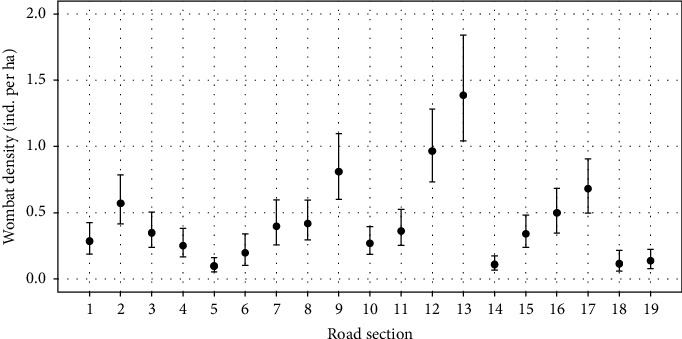
Model predictions from the final observational survey, showing the median wombat density (±95% credible interval) as individuals per hectare across the 19 road sections at Musselroe Wind Farm, Cape Portland.

Based on our wombat observation data, our model estimated a detection curve whereby wombats approximately 125 m away from the observer had a 50% probability of being detected, and animals were very unlikely to be detected when located ≥ 300 m away from the observer (Figure S3). Wombat density displayed considerable spatial variation within the study area (see Figure 1(b)). On average, wombat density was estimated to be greatest in road sections 9, 12, 13, and 17 (>0.6 wombats ha^−1^; Figure 3) and lowest in sections 5, 14, and 18 (<0.11 wombats ha^−1^; Figure 3). Mean density across the site was estimated to be 0.43 wombats ha^−1^.

**Figure 4:**
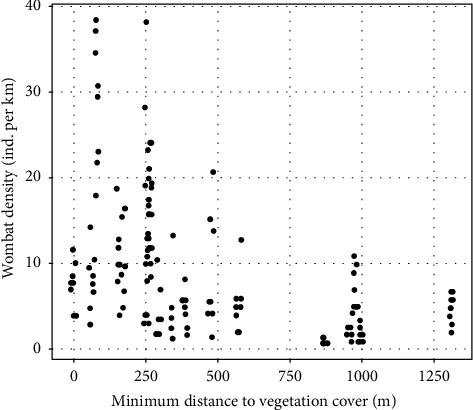
Relationship between observed wombat density and minimum distance to vegetation cover. Note that each point indicates a road section on a single observational survey and has been jittered to avoid some overlap.

Our model provided evidence of a positive temporal trend in wombat density (Table 1), with numbers predicted to have increased by a relative factor of 1.76 (95% credible interval (1.32, 2.49)) throughout the survey period (see Figure 2). We also found statistical evidence for a negative association between the minimum distance to vegetation cover and wombat density (Table 1), supporting a strong affinity of wombats to be distributed in proximity to vegetation cover (Figure 4). No significant relationships were found between wombat density and the proportion of remnant vegetation within the wombats' potential home range, nor active burrow density (Table 1).

### 3.2. Patterns and Predictors of Mange

**Figure 5:**
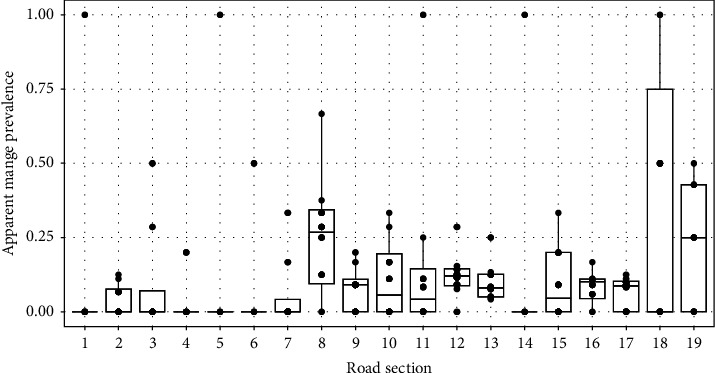
Variation in apparent mange prevalence across the 19 road sections at Musselroe Wind Farm, Cape Portland. Each point indicates the mange prevalence observed during a single observational survey. Note that some points are overlapping and may not be visible.

The mean apparent prevalence of mange was found to vary considerably across the site, ranging between 3.1 and 37.5% (Figure 5), with an overall site prevalence of 9.4%. We also found that the apparent prevalence of mange varied temporally within each road section (see Figure 5), largely driven by the small number of wombats observed within each segment on a single survey. However, we found no statistical evidence of a temporal trend in mange prevalence when considered at a site-wide level (Table 2).

**Figure 6:**
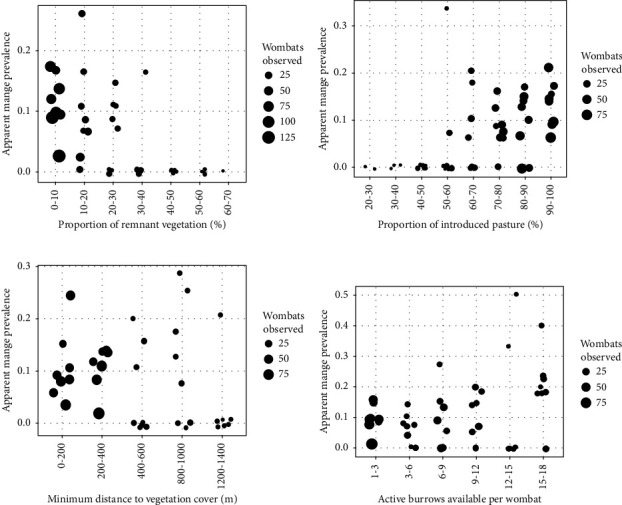
Relationship between the observed apparent prevalence of sarcoptic mange and the (a) proportion of remnant vegetation within the wombats' potential home range; (b) proportion of introduced pasture within the wombats' potential home range; (c) minimum distance to vegetation cover; and (d) active burrow to wombat ratio. Note that each point represents an observational survey, with point size indicating the number of wombats observed, and has been jittered to avoid some overlap.

Our model predictions showed a negative association between the occurrence of mange and the proportion of remnant vegetation (Table 2), with a higher apparent prevalence of mange predicted for wombats with minimal remnant vegetation within their potential home range (Figure 6(a)). However, it is important to acknowledge that the proportion of remnant vegetation was also correlated with the proportion of introduced pasture and low-lying pan, and thus these latter two features may also influence the occurrence of mange. Indeed, visual inspection of the data showed a strong positive association between mange and the proportion of introduced pasture within a wombat's potential home range (Figure 6(b)), and a weaker negative trend with low-lying pan (Figure S4). In addition, we found weak statistical evidence of a negative association between the occurrence of mange and distance to vegetation cover, and a weak positive trend between the apparent prevalence of mange and the number of active burrows available per wombat (Table 2; Figures 6(c) and 6(d)). Wombat density and active burrow density were not found to associate with the occurrence of mange (Table 2).

## 4. Discussion

Our analysis resulted in two main findings: (1) a negative association between wombat density and distance to vegetation cover and (2) a negative association between the apparent prevalence of mange and the proportion of remnant vegetation within a potential home range. We also found marginal evidence that the apparent prevalence of mange was negatively associated with distance to vegetation cover, and positively associated with the number of active burrows available per wombat. Collectively, our results suggest that wombats inhabiting less optimal habitat (i.e., introduced pasture areas, with a low proportion of remnant vegetation and low-lying pans) may be at greater risk of *S. scabiei* exposure.

### 4.1. Abundance and Geographic Distribution of Wombats

Here, we found wombats to be heterogeneously distributed across the study area, with density estimates ranging between 0.096 and 1.39 individuals ha^−1^, with these estimates broadly consistent with other larger-scale studies (e.g., see [23, 24]). For example, wombat density has been reported to range between 0.11 and 1.9 wombats ha^−1^ in eucalypt forest with adjacent pasture areas [18, 36] and 0.15–0.60 wombats ha^−1^ in agricultural areas [28, 36]. Collectively, these findings suggest that wombat occurrence is influenced by both regional and local environmental factors, and that habitat selection processes—as a function of varying resource availability and quality—likely operate across multiple spatial scales [31]. Therefore, areas associated with the lowest wombat densities at our site may reflect poorer resource quality and less optimal (sink) wombat habitat [37]. Alternatively, the low wombat density areas (e.g., introduced pasture) may reflect habitat associated with high disease risk and disease-induced mortality, and thus potentially represent an ecological trap where wombats are unable to correctly assess habitat quality and perceive it to be good when it is in fact suboptimal [38, 39]. Indeed, Roger et al. [31] suggested that wombats are frequently drawn to cleared areas and that this movement is often to their detriment. Nevertheless, further research to test these hypotheses is warranted.

Increased wombat density in areas proximate to vegetation cover is consistent with previous studies, suggesting that vegetation (e.g., boxthorn, tussock grass, and native shrub) provides favorable burrowing conditions (e.g., well-draining soil and resilience to collapse) and refuge from abiotic and biotic threats [28, 31, 40], as wombat home ranges are closely associated with the location of their burrows [24]. This finding also suggests that burrows situated in open pasture may be less optimal, as they are more vulnerable to collapse and flooding, and may thus be actively avoided by wombats. We did not find any association between wombat density and active burrow density, nor the proportion of remnant vegetation within the road segments, suggesting that the animals are burrowing in the dense vegetation surrounding the site (outside our burrow density survey areas) and are moving into the open pasture (observable area) to forage at night. This is broadly consistent with other literature on the behavior and ecology of bare-nosed wombats (e.g., see [24, 28]).

The marked increase in wombat abundance we observed across our surveys was unexpected based on previous research conducted in the area (see [22]) and cannot be attributed to within-site reproduction alone given typical wombat reproductive rates (see [41]). Rather, the increase in numbers may be due to increased local migration from the dense vegetation surrounding the site, possibly attributed to seasonal changes in resource availability, day length, and wombat diel activity patterns during the survey period [18, 24, 42]. Indeed, Stannard et al. [23] reported a similar seasonal increase at Eagle's Drift, New South Wales in 2017, with wombat numbers increasing by a factor of 3.67 from autumn to winter and 1.82 from winter to spring. Interestingly, these seasonal changes in wombat observations coincide with Australian roadkill records—with wombat-vehicle collisions peaking during winter and early-spring [43]—and may suggest that wombats are “on the move” more during these periods. Furthermore, a recent reduction in the number of crop protection permits, that sanction the killing of wombats, in the surrounding areas of northeast Tasmania may have contributed to regional population growth and thus increased movement into the study area [44].

### 4.2. Apparent Prevalence, Patterns, and Predictors of Mange

We found wombats showing visible signs of mange to be widespread across the site, occurring at a mean apparent prevalence of 9.4%. This finding is consistent with what might be expected for an endemic pathogen and concurs with previous research from the area (8.8–13.7% prevalence) [22]. Furthermore, we found the mean apparent prevalence of mange to vary considerably across the site, ranging between 3.1 and 37.5% within the 19 road segments. This level of variation is comparable to that observed at a regional scale [22, 23] and provides evidence that spatiotemporal patterns of disease can manifest at surprisingly fine spatial scales and within relatively homogenous environments, which are often overlooked despite the seemingly obvious implications for management [5].

Our finding that wombats are more likely to have mange when remnant vegetation comprises a lower proportion of their potential home range helps advance understanding of the landscape epidemiology of this disease. However, the mechanistic cause of this association is not yet entirely clear. Remnant vegetation may act as a microclimatic buffer, maintaining warmer and drier within-burrow conditions [45, 46] that are less conducive for off-host *S. scabiei* survival in the environment, and thus transmission. Alternatively, as remnant vegetation appears to positively influence wombat habitat selection—suggestive of higher habitat quality—individuals inhabiting areas comprised of greater remnant vegetation may be of better health, able to competitively exclude unhealthy wombats from more optimal burrows, and may be less susceptible to infection [47]. Seemingly in contrast, we also found a trend that individuals proximate to vegetation cover (although not occurring within their potential home range) were more likely to show visible signs of mange. However, this trend may reinforce the idea that mange-affected wombats may be competitively excluded from burrowing in the dense vegetation and are forced to occupy less optimal burrows in the immediately surrounding pasture areas. It is also possible that this trending relationship suggests that while mange-affected individuals typically have a low proportion of remnant vegetation within their potential home range, they are unable to migrate far from their burrows to forage on pasture and were thus observed near the surrounding vegetation cover. More broadly, the associations between mange exposure and vegetation in this study are mirrored by regional-scale research by Driessen, Dewar, Carver, and Gales [22], who found mange to be disproportionately common in “agricultural, urban, and exotic” vegetation communities and comparatively uncommon in native landscapes despite an abundance of wombats in these habitat groups. Further research on the mechanisms underlying the mange prevalence and vegetation associations is warranted, as well as the potential for revegetation as a mange mitigation strategy (see final paragraph).

Contrary to expectations, we found a trending positive association between the apparent prevalence of mange and the number of active burrows available per wombat. This result is surprising, as models predict greater pathogen transmission when there are fewer burrows available per wombat, such that wombats “share” burrows more frequently [17]. We propose several hypotheses that may explain this result. First, the regions associated with a higher apparent prevalence of mange (i.e., introduced pasture) are expected to be associated with an increased disease-induced mortality rate [37]. Thus, the possible association between mange and active burrows available per wombat may be an artifact of increased wombat mortality in these areas. Alternatively, a greater availability of active burrows may enable mange-affected wombats to switch burrows more frequently, thus increasing the number of contaminated burrows within the landscape and the potential for a susceptible individual to enter a contaminated burrow. Third, while less active burrows available per wombat in the lower mange prevalence areas may suggest that they are more optimal and nearing complete occupancy, our previous results suggest a large proportion of the wombats are burrowing in the dense vegetation in the surrounding areas of the site, which are slightly outside of the target burrow density survey areas in this study. Therefore, it is possible that our burrow density quadrats did not capture all the most relevant burrows for wombats and our estimates of active burrow to wombat ratio may be conservative. Of a similar nature, this may explain why active burrow density was not found to influence the occurrence of mange despite burrows being well accepted as the site responsible for most transmission events [12, 17]. Finally, while somewhat expected for an indirectly transmitted pathogen [48], we found no evidence for an effect of wombat density on mange prevalence, indicating that transmission can be maintained independently of host density within this system. Nevertheless, it would be of interest to further examine this relationship at other sites with varying disease dynamics and landscape attributes.

This study has potential implications for the design and implementation of effective disease management interventions, which may be cross-applicable to other host species with indirect transmission through shared environments, such as the American black bear (*Ursus americanus*) and San Joaquin kit fox (*Vulpes macrotis mutica*) [12]. For example, our model results suggest that management efforts and resources may be most beneficial if focused in exotic and modified landscapes (e.g., agricultural areas), which appear to be less optimal for wombats, conducive for off-host mite survival, and contribute to a disproportionate number of mange cases [22]. In addition, our results highlight the importance of remnant vegetation for predicting the occurrence of mange and propose the idea of revegetation as a potential disease mitigation strategy, although further research in this area is warranted. This study also provides evidence that spatiotemporal patterns of mange can manifest at surprisingly fine spatial scales, even within relatively homogenous environments, and thus highlights the potential benefit of constructing disease distribution maps and identifying small regions of focus for targeted disease management and parasiticide treatment efforts. Furthermore, factors identified to be associated with the occurrence of mange (e.g., a low proportion of remnant vegetation) may be projected to other geographical regions or times and provide an *a priori* understanding of disease risk and/or spread [3]. Finally, and more broadly, our results contribute to a deeper understanding of the landscape epidemiology of sarcoptic mange and fine-scale factors driving endemic disease dynamics.

## Figures and Tables

**Table 1:** Parameter estimates and their uncertainty associated with the model used to predict wombat density.

Predictors	Coefficient	2.5% CI	97.5% CI
Survey date	1.116	0.546	1.807
Minimum distance to vegetation cover	−0.453	−0.837	−0.050
Proportion of remnant vegetation	0.163	−0.264	0.607
Active burrow density	−0.098	−0.562	0.319

Estimates are based on *z*-transformed predictors so are dimensionless and comparable. CI denotes credible interval.

**Table 2:** Parameter estimates and their uncertainty associated with the model used to predict sarcoptic mange in wombats.

Predictors	Coefficient	2.5% CI	97.5% CI
Survey date	−1.228	−2.989	0.566
Minimum distance to vegetation cover	−0.256	−0.593	0.082
Proportion of remnant vegetation	−0.501	−0.862	−0.173
Active burrow density	−0.237	−0.755	0.280
Wombat density	0.050	−0.467	0.525
Active burrow to wombat ratio	0.533	−0.095	1.094

Estimates are based on *z*-transformed predictors so are dimensionless and comparable. CI denotes credible interval.

## Data Availability

The data that support the findings of this study are available from the corresponding author upon reasonable request.

## Supplementary Materials

Supplementary Materials
*Supplementary 1*. Table S1: Mange severity scoring system used to classify a wombat's mange status, adapted from the study by Simpson et al. [11]. *Supplementary 2*. Figures S1A–B: Satellite image of the study area showing (A) the geographical location of the 60 burrow density quadrats and (B) the minimum straight-line distance to dense vegetation cover, measured as a straight line from the midpoint of each road section. *Supplementary 3*. Figures S2A–B: (A) Satellite image showing the extent of the study area visible in panel B. (B) Feature layer with polygons illustrating eight identifiable landscape features. *Supplementary 4*. Figure S3: The half-normal detection curve used to estimate wombat density, relative to the perpendicular distance from the observer. *Supplementary 5.* Figure S4: Relationship between the observed apparent prevalence of mange and the proportion of low-lying pan within a wombat's potential home range.
