# Supplementary material for: Fine-Scale Landscape Epidemiology: Sarcoptic Mange in Bare-Nosed Wombats (Vombatus ursinus)
Source: Transbound Emerg Dis. 2023 Mar 4;2023:2955321. doi: 10.1155/2023/2955321 (PMC12016856; doi:10.1155/2023/2955321)
Supplement: Supplementary Materials — Supplementary 1. Table S1: Mange severity scoring system used to classify a wombat's mange status, adapted from the study by Simpson et al. [11]. Supplementary 2. Figures S1A–B: Satellite image of the study area showing (A) the geographical location of the 60 burrow density quadrats and (B) the minimum straight-line distance to dense vegetation cover, measured as a straight line from the midpoint of each road section. Supplementary 3. Figures S2A–B: (A) Satellite image showing the extent of the study area visible in panel B. (B) Feature layer with polygons illustrating eight identifiable landscape features. Supplementary 4. Figure S3: The half-normal detection curve used to estimate wombat density, relative to the perpendicular distance from the observer. Supplementary 5. Figure S4: Relationship between the observed apparent prevalence of mange and the proportion of low-lying pan within a wombat's potential home range. [file 2955321.f1.zip › Figure S3.docx]

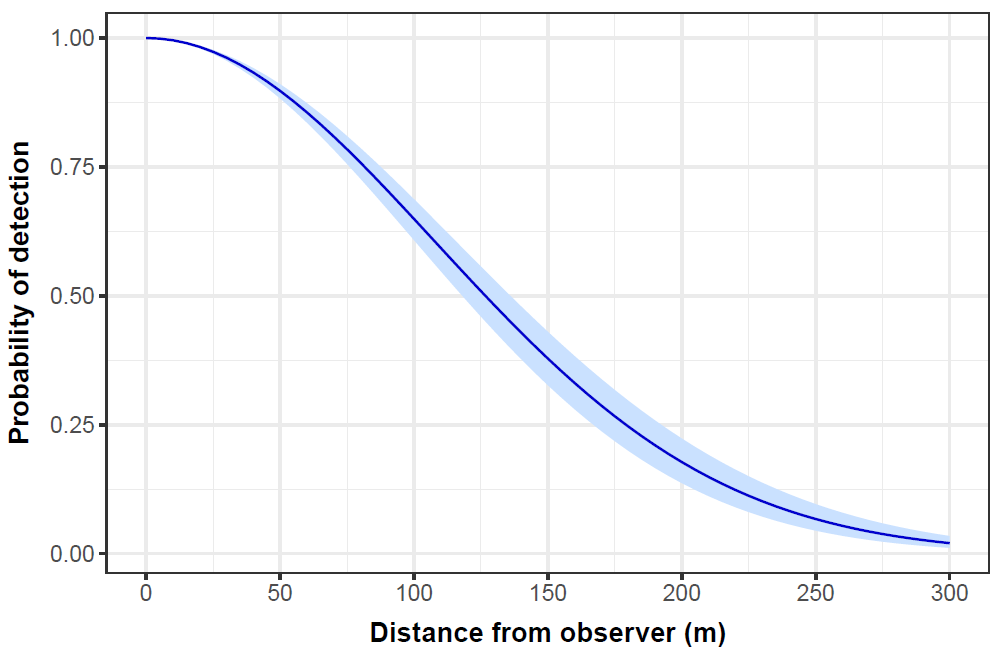
**Figure S3:** The half-normal detection curve used to estimate wombat density, relative to the perpendicular distance from the observer. Inner dark blue line indicates the median detection probability, with the outer light blue ribbon showing the associated 95% credible interval.
