# Supplementary material for: Fine-Scale Landscape Epidemiology: Sarcoptic Mange in Bare-Nosed Wombats (Vombatus ursinus)
Source: Transbound Emerg Dis. 2023 Mar 4;2023:2955321. doi: 10.1155/2023/2955321 (PMC12016856; doi:10.1155/2023/2955321)
Supplement: Supplementary Materials — Supplementary 1. Table S1: Mange severity scoring system used to classify a wombat's mange status, adapted from the study by Simpson et al. [11]. Supplementary 2. Figures S1A–B: Satellite image of the study area showing (A) the geographical location of the 60 burrow density quadrats and (B) the minimum straight-line distance to dense vegetation cover, measured as a straight line from the midpoint of each road section. Supplementary 3. Figures S2A–B: (A) Satellite image showing the extent of the study area visible in panel B. (B) Feature layer with polygons illustrating eight identifiable landscape features. Supplementary 4. Figure S3: The half-normal detection curve used to estimate wombat density, relative to the perpendicular distance from the observer. Supplementary 5. Figure S4: Relationship between the observed apparent prevalence of mange and the proportion of low-lying pan within a wombat's potential home range. [file 2955321.f1.zip › Figure S4 (1).docx]

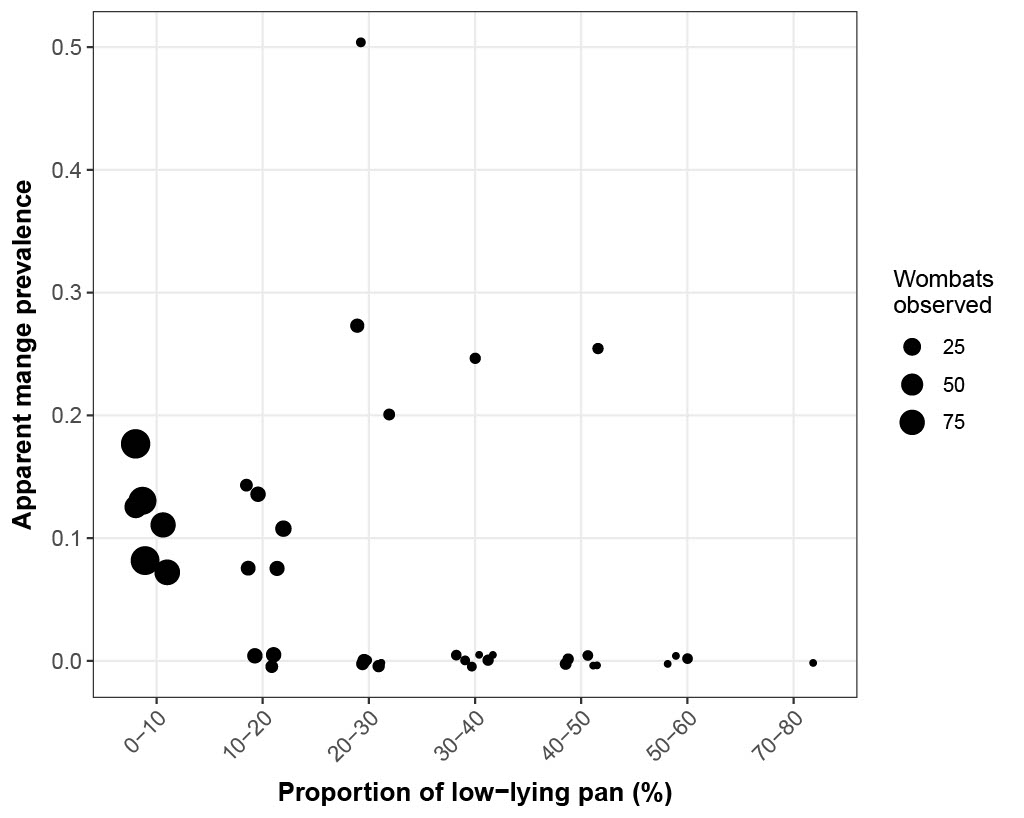


**Figure S4**: Relationship between the observed apparent prevalence of mange (i.e., proportion of wombats showing clinical signs of disease) and the proportion of low-lying pan within a wombat’s potential home range. Each point represents a survey trip with point size indicating the number of wombats observed. Note that the points have been jittered to avoid some overlap.
