# Supplementary material for: Fine-Scale Landscape Epidemiology: Sarcoptic Mange in Bare-Nosed Wombats (Vombatus ursinus)
Source: Transbound Emerg Dis. 2023 Mar 4;2023:2955321. doi: 10.1155/2023/2955321 (PMC12016856; doi:10.1155/2023/2955321)
Supplement: Supplementary Materials — Supplementary 1. Table S1: Mange severity scoring system used to classify a wombat's mange status, adapted from the study by Simpson et al. [11]. Supplementary 2. Figures S1A–B: Satellite image of the study area showing (A) the geographical location of the 60 burrow density quadrats and (B) the minimum straight-line distance to dense vegetation cover, measured as a straight line from the midpoint of each road section. Supplementary 3. Figures S2A–B: (A) Satellite image showing the extent of the study area visible in panel B. (B) Feature layer with polygons illustrating eight identifiable landscape features. Supplementary 4. Figure S3: The half-normal detection curve used to estimate wombat density, relative to the perpendicular distance from the observer. Supplementary 5. Figure S4: Relationship between the observed apparent prevalence of mange and the proportion of low-lying pan within a wombat's potential home range. [file 2955321.f1.zip › Table S1.docx]

| **Mange score** | **Score description** | **Mange severity status** |
| --- | --- | --- |
| X | Wombat not assessed for mange | Unknown |
| 0 | No signs of mange observed on segment | Healthy |
| 1 | Ambiguous, possible hair thinning | Likely healthy |
| 2 | <10% of body affected by mange | Early mange |
| 3 | 10–40% of body affected by mange | Moderate mange |
| 4 | 40–60% of body affected by mange | Severe mange |
| 5 | >60% of body affected by mange | Late-stage mange (terminal) |

**Table S1:** Mange severity scoring system used to classify a wombat’s mange status (adapted from: Simpson et al., 2016).

Note: For the purpose of analysis, wombats with a score of 0 or 1 were classified as ‘healthy’ and those with a score between 2 and 5 were classified as ‘mange affected’.
